# Induced superconducting correlations in a quantum anomalous Hall insulator

**DOI:** 10.1038/s41567-024-02574-1

**Published:** 2024-07-10

**Authors:** Anjana Uday, Gertjan Lippertz, Kristof Moors, Henry F. Legg, Rikkie Joris, Andrea Bliesener, Lino M. C. Pereira, A. A. Taskin, Yoichi Ando

**Affiliations:** 1 Physics Institute II, University of Cologne, Cologne, Germany; 2 Quantum Solid State Physics, KU Leuven, Leuven, Belgium; 3 Peter Grünberg Institute (PGI-9), Forschungszentrum Jülich & JARA-Fundamentals of Future Information Technology, Jülich-Aachen Research Alliance, Jülich, Germany; 4 Department of Physics, University of Basel, Basel, Switzerland

**Keywords:** Topological insulators, Superconducting properties and materials, Quantum Hall, Superconducting devices

## Abstract

Thin films of ferromagnetic topological insulator materials can host the quantum anomalous Hall effect without the need for an external magnetic field. Inducing Cooper pairing in such a material is a promising way to realize topological superconductivity with the associated chiral Majorana edge states. However, finding evidence of the superconducting proximity effect in such a state has remained a considerable challenge due to inherent experimental difficulties. Here we demonstrate crossed Andreev reflection across a narrow superconducting Nb electrode that is in contact with the chiral edge state of a quantum anomalous Hall insulator. In the crossed Andreev reflection process, an electron injected from one terminal is reflected out as a hole at the other terminal to form a Cooper pair in the superconductor. This is a compelling signature of induced superconducting pair correlation in the chiral edge state. The characteristic length of the crossed Andreev reflection process is found to be much longer than the superconducting coherence length in Nb, which suggests that the crossed Andreev reflection is, indeed, mediated by superconductivity induced on the quantum anomalous Hall insulator surface. Our results will invite future studies of topological superconductivity and Majorana physics, as well as for the search for non-abelian zero modes.

## Abstract (web summary)

The superconducting proximity effect has not been experimentally demonstrated in a quantum anomalous Hall insulator. Now this effect is observed in the chiral edge state of a ferromagnetic topological insulator.

## Main

**Fig. 1:**
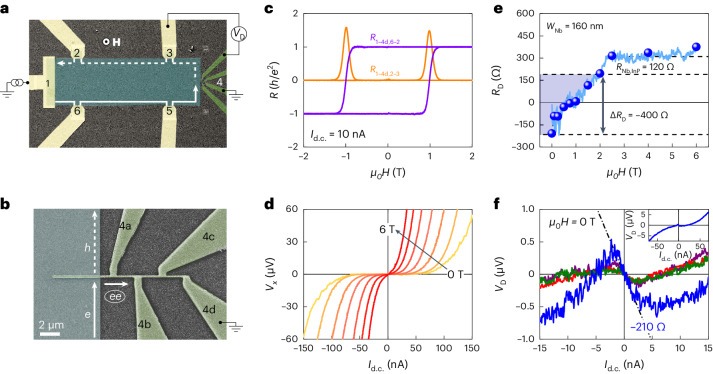
CAR across the quantum anomalous Hall edge state. **a**, False-colour scanning electron microscopy image of device A including the measurement schematics. The SC Nb electrode (green) and the Ti/Au normal electrodes (yellow) are in contact with the V-doped (Bi_*x*_Sb_1−*x*_)_2_Te_3_ thin film (cyan). For an upward, out-of-plane magnetization (*M* > 0), the chiral 1D edge state propagates anticlockwise along the sample edge. The voltage *V*_D_ between contacts 3 and 4 gives the downstream resistance *R*_D_ ≡ *V*_D_/*I*_d.c._. **b**, Magnified image of the 160-nm-wide Nb electrode shown in **a**. The white arrows schematically show the CAR process. **c**, Magnetic-field dependence of the four-terminal resistances, showing the QAHE with vanishing longitudinal resistance *R*_1–4d,2–3_ = 0 and quantized transverse resistances *R*_1–4d,6–2_ = *h*/*e*^2^ at 25 mK. **d**, Current versus voltage (*I*–*V*) characteristics of the four-terminal longitudinal voltage *V*_*x*_ at 17 mK in various applied magnetic fields *H* from 0 to 6 T in steps of 1 T. The breakdown current decreases with increasing *H*. **e**, The light blue line shows the downstream resistance *R*_D_ continuously measured as a function of *H* from 0 to 6 T with *I*_d.c._ = 2 nA at 25 mK. Blue symbols represent the slopes of the *I*–*V* characteristics at *I*_d.c._ = 0 at discrete magnetic fields (Supplementary Note 3), which give confidence in the negative *R*_D_ indicative of CAR. As the superconductivity in Nb is suppressed with increasing *H*, *R*_D_ increases by 520 Ω, which consists of the normal-state Nb resistance (120 Ω, marked by a dashed line) and the CAR contribution Δ*R*_D_ ≃ −400 Ω (marked by blue shading). The *R*_D_ level of ~180 Ω marked by the middle horizontal dashed line corresponds to *R*_contact_, which gives a positive offset to the raw *R*_D_. **f**, *I*–*V* curves for the downstream voltage *V*_D_ measured in 0 T at 17 mK for different magnetic-field-sweep histories. The magnitude of the negative slope at *I*_d.c._ = 0 depends on the history. See Supplementary Note 4 for details. Inset, An *I*–*V* curve up to ±70 nA dominated by the current-induced breakdown of the QAHE. Source data

Inducing superconducting (SC) correlations using the SC proximity effect in the one-dimensional (1D) edge state of a two-dimensional (2D) topological system would lead to exotic topological superconductivity hosting non-abelian anyons^1–7^ and, hence, has been experimentally pursued in a couple of systems. For the 1D helical edge state of a 2D topological insulator (TI), the induced SC correlations have been detected in Josephson junctions^8,9^. The SC correlations in the quantum Hall edge states are less trivial due to the chiral nature of the edge and large magnetic fields required, but strong evidence has been obtained in terms of the crossed Andreev reflection (CAR)^10–13^ or the formation of Andreev edge states^14–16^, which cause a negative nonlocal potential in the downstream edge^17–20^. In the CAR process, an electron in the chiral edge entering a grounded SC electrode creates a Cooper pair by taking another electron from the other side of the electrode, causing a hole to exit into the downstream edge (Fig. 1b). This hole is responsible for the negative nonlocal voltage observed experimentally^17,20^. Importantly, SC correlations are induced in the chiral edge state through the CAR process. Very recently, the CAR process has been observed even in the fractional quantum Hall edge states^20^, which are an interesting platform for creating parafermions obeying rich non-abelian statistics^21,22^.

In this context, the SC proximity effect in a quantum anomalous Hall insulator (QAHI), which is a ferromagnetic TI showing the quantum anomalous Hall effect (QAHE), is highly interesting. If the 1D edge state of a QAHI can be proximitized, one could create a non-abelian Majorana zero mode by coupling two counter-propagating edges by the CAR process through a superconductor^6,10,17^. If, on the other hand, the 2D surface of the QAHI is proximitized, a chiral Majorana edge state may occur^2,23^, which could be a platform for flying topological qubits that transfer information between stationary qubits^24–27^. Hence, proximitized QAHI is an interesting platform for Majorana physics. However, no clear evidence has been reported for the SC proximity effect in a QAHI^28–30^.

The QAHI can be realized by doping Cr or V into a very thin film (typically ≲10 nm thickness) of the three-dimensional TI material (Bi_*x*_Sb_1−*x*_)_2_Te_3_ in which the chemical potential is fine-tuned into a magnetic gap that opens at the Dirac point of the surface states as a result of a ferromagnetic order^31–33^. Hence, a QAHI is insulating, not only in the three-dimensional bulk but also in the 2D surface. Inducing SC correlations in bulk-insulating TIs is much more difficult than in bulk-conducting TIs^34^, and this is one of the reasons for the lack of clear evidence for the SC proximity effect in a QAHI. In fact, a recent work reported the observation of Andreev reflection in a metallic regime of a magnetic TI film, but when the sample was in the QAHI regime, there was no evidence for any Andreev process^28^. Another work in this context^29^ used a device structure that was not optimal for detecting the relevant Andreev process. Recent experiments on quantum Hall systems found a robust signature of CAR even at the spin-polarized *ν* = 1 filling factor^17,20^, which appears to resemble a QAHI edge. However, an important difference is that a QAHI edge is not fully spin polarized^35^. In the present work, we have successfully observed the signature of CAR with a narrow Nb finger electrode (down to 160 nm width) in contact with the QAHI edge. The finger-width dependence of the CAR signal gives the characteristic length of the CAR process that is much longer than the SC coherence length of Nb, which suggests that it is not the superconductivity in the Nb electrode but the proximity-induced pairing in the QAHI beneath the Nb that is mediating the CAR process.

## Nonlocal detection of CAR

Our samples are Hall-bar devices of V-doped (Bi_*x*_Sb_1−*x*_)_2_Te_3_ (ref. ^36^) in contact with SC Nb electrodes with widths ranging from 160 to 520 nm. Figure 1a,b shows false-colour scanning electron microscopy images of device A, which had the narrowest Nb electrode (contact 4). All other contacts were made of Ti/Au with contact resistances of a few ohms (Supplementary Note 1). The 1D chiral edge state propagates in the anticlockwise direction for an upward, out-of-plane magnetization (*M* > 0). For the configuration shown in Fig. 1a, we set a d.c. current to flow between contacts 1 and 4d; namely, a voltage was applied to the normal metal contact 1 and the SC contact was grounded.

In ref. ^28^, Andreev reflections of the electrons in the 2D ‘bulk’ states of a magnetic TI film in the metallic regime were observed in devices like the one in Fig. 1a, but here we probe the SC correlations in the 1D chiral edge state of the QAHI. For our purpose, confirmation of the dissipationless edge transport without the contribution of the 2D bulk is essential. In fact, the longitudinal resistance *R*_*xx*_ (= *R*_1–4d,2–3_ measured between contacts 2 and 3 with the current between 1 and 4d) vanishes in our devices, whereas the transverse resistance *R*_*yx*_ (= *R*_1–4d,6–2_ measured between contacts 6 and 2) is quantized to *h*/*e*^2^, where *h* is the Planck’s constant and *e* is the elementary charge, without the need for electrostatic gating, as shown in Fig. 1c. Note that a breakdown of the zero-resistance state occurs when the current exceeds a critical current^36–42^, and the zero-resistance region is observed to shrink with increasing magnetic field, as shown in Fig. 1d, which is possibly caused by a charge redistribution between the bulk and the QAHI edge in applied magnetic fields^43,44^. This fragility of the QAHI state against current makes it difficult to estimate the contact transparency using current biasing^17,28^.

The CAR process converts an incoming electron with an energy *eV* that is smaller than the SC gap *Δ* into a hole carrying a potential of −*V* in the downstream edge (Fig. 1b), which is detected at contact 3 as the downstream voltage *V*_D_ with respect to the grounded SC contact 4a. Here, downstream refers to the chiral direction of the edge state (Fig. 1a,b). In addition, there is a finite probability that an upstream electron will tunnel directly into the downstream as an electron carrying a positive potential *V*. This is called co-tunnelling (CT), and it competes with the CAR process in the nonlocal transport^11–13^. The downstream resistance *R*_D_ ≡ *V*_D_/*I*_d.c._ observed in this configuration consists of

1 $${R}_{{{{\rm{D}}}}}={R}_{{{{\rm{QAHI}}}}}+{R}_{{{{\rm{Nb,InP}}}}}+{R}_{{{{\rm{contact}}}}}+{R}_{{{{\rm{D}}}}}^{{{{\rm{i}}}}},$$

where the resistance of the QAHI film *R*_QAHI_ is zero for low probe currents below the breakdown, *R*_Nb,InP_ is the resistance of the Nb section lying on the InP wafer between the film edge and the SC contact 4a (which is zero when the Nb is SC), *R*_contact_ is the extrinsic contact resistance due to the imperfect Nb–QAHI interface and ${R}_{{{{\rm{D}}}}}^{{{{\rm{i}}}}}$ is the intrinsic downstream resistance reflecting the CAR/CT contribution. The subgap states in the SC due to, for example, vortices can provide a dissipative channel that dumps electrons to the ground, which will reduce ${R}_{{{{\rm{D}}}}}^{{{{\rm{i}}}}}$ (refs. ^17–19^). Note that the present set-up is a three-terminal configuration and that *R*_contact_ always gives a finite contribution to *V*_D_. An external magnetic field is not required for the realization of the QAHE, enabling us to examine ${R}_{{{{\rm{D}}}}}^{{{{\rm{i}}}}}$ as a function of the applied magnetic field from 0 T up to the upper critical field *H*_c2_ of SC Nb. This is an important difference from previous studies of the SC proximity effect in quantum Hall edge states^17–20^. The magnetization measurements of our QAHI films found that the magnetic induction produced by the ferromagnetism of the film was only ~4 mT in a near-zero applied magnetic field at 2 K (Supplementary Note 13). This is smaller than the lower critical field of Nb (~180 mT)^45^ and would not create vortices, which harbour subgap states and allow incident electrons to be dissipated without the Andreev mechanism^17,46,47^. However, one cannot exclude the possibility that some vortices remain trapped at strong pinning centres. Due to the chiral nature of the edge state, no Andreev reflection occurs into the upstream edge.

Figure 1e shows the magnetic-field dependence of *R*_D_ for device A with a Nb electrode of width *W*_Nb_ = 160 nm, measured with current *I*_d.c._ = 2 nA (see Supplementary Notes 3 and 4 for additional data). Below ~1 T, the downstream resistance is negative, signalling the CAR process across the Nb electrode. This is the main result of this work and demonstrates that SC correlations are induced in the chiral edge state across the SC finger by CAR processes in our devices. As the magnetic field is increased, *R*_D_ gradually turns positive and saturates as the superconductivity is lost in the Nb electrode. The change in the nonlocal downstream resistance due to the suppression of the CAR/CT process is calculated as Δ*R*_D_ ≡ −[*R*_D_(*H* > *H*_c2_) − *R*_D_(*H* < *H*_c2_) − *R*_Nb,InP_], which should be equal to ${R}_{{{{\rm{D}}}}}^{{{{\rm{i}}}}}$ provided that *R*_QAHI_ remains zero and *R*_contact_ does not change across *H*_c2_ (which we confirmed in wide-finger devices; see Supplementary Note 14). When Δ*R*_D_ is negative (positive), the CAR (CT) process is dominant. We estimate Δ*R*_D_ ≈ −400 Ω after subtracting the contribution of the normal-state Nb resistance *R*_Nb,InP_ ≈ 120 Ω (Fig. 1e and Supplementary Note 2). As ${R}_{{{{\rm{D}}}}}^{{{{\rm{i}}}}}$ = 0 in the normal state, *R*_D_ = *R*_Nb,InP_ + *R*_contact_ holds at *H* > *H*_c2_ and below the breakdown current, allowing us to evaluate *R*_contact_ and conclude that the CAR process contributes ${{\Delta }}{R}_{{{{\rm{D}}}}}(={R}_{{{{\rm{D}}}}}^{{{{\rm{i}}}}})\approx -400\,{{\Omega }}$, which is much larger than the measured negative *R*_D_. This Δ*R*_D_ corresponds to about 3% of the maximum negative downstream resistance −*h*/2*e*^2^ expected for 100% CAR (Supplementary Note 8).

**Fig. 2:**
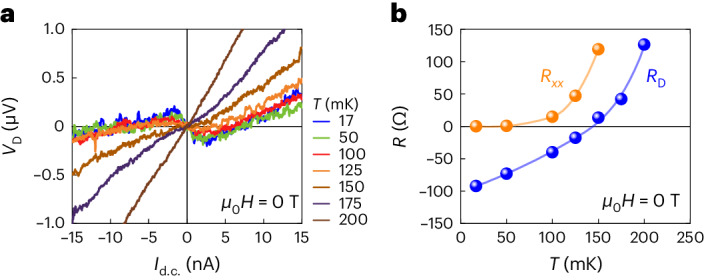
Temperature dependence of the downstream potential in device A. **a**, Plots of *V*_D_ versus *I*_d.c._ at different temperatures measured with the set-up shown in Fig. 1a. **b**, Temperature dependencies of *R*_D_, extracted from the *I*–*V* curves in **a** at *I*_d.c._ = 0 (blue), and the four-terminal longitudinal resistance *R*_*xx*_ (orange). Above 50 mK, *R*_*xx*_ deviates from zero, indicating that the dissipationless transport of the QAHE is lost. Consequently, the 2D bulk resistance eventually dominants *R*_D_ at *T* ≳ 100 mK. Source data

To give confidence that the negative *R*_D_ is not just a result of voltage fluctuations, the *I*–*V* characteristics for the downstream voltage *V*_D_ in 0 T are shown in Fig. 1f. The slope in the zero-current limit (which also gives *R*_D_) is reproducibly negative for all the measured curves for different magnetic histories, even though the magnitude of *R*_D_ changes with the magnetic history (Supplementary Note 5), which was probably caused by a change in the disorder profile. The small nonreciprocity seen in Fig. 1f is due to 1D chiral edge transport itself^48,49^. At high current, the breakdown of the QAHE (causing *R*_QAHI_ > 0) dominates the downstream voltage (Fig. 1f, inset). The change in the behaviour of *V*_D_ versus *I*_d.c._ with increasing temperature is shown in Fig. 2a. The *R*_D_ values extracted from these data are plotted in Fig. 2b as a function of temperature along with the four-terminal longitudinal resistance *R*_*xx*_, which starts to deviate from zero above ~50 mK, behaviour typical of the QAHI samples available today^36–40^. Obviously, the CAR contribution in *R*_D_ is masked by the contribution of *R*_QAHI_ at *T* > 50 mK. This observation demonstrates a clear link between the negative *R*_D_ and the QAHI edge transport.

## Finger-width dependences of the downstream resistance

**Fig. 3:**
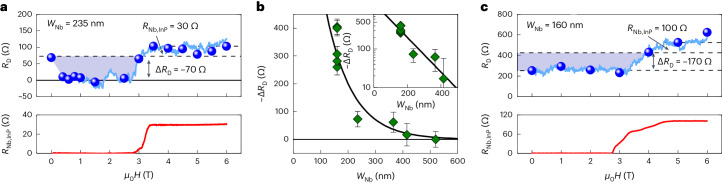
Dependencies of CAR on the width and interface quality. **a**, Magnetic-field dependence of *R*_D_ for the 235-nm-wide Nb electrode of device B shown together with *R*_Nb,InP_. The light blue line shows the *R*_D_ continuously measured in a magnetic-field sweep at 25 mK with *I*_d.c._ = 2 nA. Blue symbols represent the slopes of the *I*–*V* curves at *I*_d.c._ = 0. The *R*_D_ level without *R*_Nb,InP_ is marked by a dashed line. The change in the downstream resistance due to CAR, Δ*R*_D_, is estimated to be about −70 Ω in this sample. **b**, Exponential width dependence of Δ*R*_D_. Green symbols correspond to the data for devices A–E fabricated on the same wafer. The calculations of Δ*R*_D_ for five different magnetic histories in device A are explained in Supplementary Note 5. The Δ*R*_D_ values for devices B–E were obtained from the data shown in **a** and in Supplementary Fig. 7. The error bars are not due to statistics but represent uncertainties discussed in Supplementary Notes 14 and 15. Inset, The same data on a semi-log plot. The solid black line is a fit of the data to ${{\Delta }}{R}_{{{{\rm{D}}}}}={R}_{0}\exp (-{W}_{{{{\rm{Nb}}}}}/{\xi }_{{{{\rm{CAR}}}}})$, yielding *R*_0_ ≈ −750 Ω and *ξ*_CAR_ ≈ 100 nm. **c**, Similar measurement as in **a** for the 160-nm-wide Nb electrode of device F, fabricated on a different wafer a few months after film growth. Reflecting the relatively large interface resistance between the QAHI film and the Nb electrode, the *R*_D_ of device F was ~255 Ω, even when the Nb was SC, and it increased to ~525 Ω above *H*_c2_. Nevertheless, Δ*R*_D_ ≃ −170 Ω was still obtained for this 160-nm-wide Nb electrode. Source data

We further investigated *R*_D_ for devices with different Nb finger widths up to 520 nm. The magnetic-field dependence of *R*_D_ in device B with *W*_Nb_ = 235 nm is shown in Fig. 3a (see Supplementary Note 7 for data on devices C–E, which had wider fingers). The estimated Nb finger resistance *R*_Nb,InP_ is also shown for comparison. Notice that the increase in *R*_D_ coincided with the suppression of the superconductivity in Nb. The *R*_D_ value fluctuated around zero in this *W*_Nb_ = 235 nm sample when the Nb was SC, which indicates that the negative CAR contribution (${R}_{{{{\rm{D}}}}}^{{{{\rm{i}}}}}$) happened to be nearly of the same magnitude as *R*_contact_, so that the resulting *R*_D_ was around zero. Note that simple Andreev reflection can account for only a factor of 2 reduction in the interface resistance^28,50^ and cannot explain why *R*_D_ went to zero. We estimated Δ*R*_D_ = −70 Ω for device B (Fig. 3a). In addition, we confirmed that *R*_U_ − *R*_D_ = *h*/*e*^2^, where *R*_U_ is the upstream resistance, which must hold if Δ*R*_D_ is due to Andreev processes whose contribution should cancel in *R*_U_ − *R*_D_ (Supplementary Notes 8 and 12). Note that not only was *R*_D_ < 0 observed for device A but also that *R*_D_ = 0 was observed for devices B (Fig. 3a) and C (*W*_Nb_ = 365 nm; Supplementary Fig. 7a), which cannot be understood without CAR. Hence, the existence of CAR for *W*_Nb_ up to 365 nm can be inferred from the raw *R*_D_ data without analysis.

For comparison, we show in Fig. 3c the data for a *W*_Nb_ = 160 nm sample (device F), which was fabricated several months after the film was grown. The ageing of the film caused a large *R*_contact_, and the CAR contribution ${{\Delta }}{R}_{{{{\rm{D}}}}}(={R}_{{{{\rm{D}}}}}^{{{{\rm{i}}}}})$ could not make *R*_D_ become negative or zero, even though the width of this sample was the same as that of device A. Using the estimated *R*_Nb,InP_ ≃ 100 Ω, we obtained *R*_contact_ ≃ 420 Ω and Δ*R*_D_ ≃ −170 Ω for this device F, pointing to the robustness of the CAR process even for a poor contact. Note that devices A–E were of higher quality because they were fabricated on a fresh QAHI film immediately after the growth.

As summarized in Fig. 3b, a finite negative Δ*R*_D_ was obtained up to *W*_Nb_ ≃ 500 nm. For device A (*W*_Nb_ = 160 nm), as already mentioned, different values of the negative *R*_D_(*H* < *H*_c2_) were obtained for different magnetic-field sweeps due to the changing disorder profiles. These are included in Fig. 3b as individual data points (see Supplementary Note 5 for the calculations of the Δ*R*_D_ values). One can see in the inset of Fig. 3b that, on average, the magnitude of Δ*R*_D_ was exponentially suppressed with increasing *W*_Nb_. A fit to ${{\Delta }}{R}_{{{{\rm{D}}}}}={R}_{0}\exp (-{W}_{{{{\rm{Nb}}}}}/{\xi }_{{{{\rm{CAR}}}}})$ gives *R*_0_ ≈ −750 Ω and the characteristic length of the CAR process *ξ*_CAR_ ≈ 100 nm. This is much longer than the SC coherence length of dirty Nb, that is $\sqrt{{\xi }_{{{{\rm{BCS}}}}}{l}_{{{{\rm{mfp}}}}}}\approx 30$ nm, with the BCS coherence length ${\xi}_{\rm{BCS}}={\hslash} {v}_{\mathrm{F}}^{\mathrm{S}}/{\uppi} {\Delta}$, Fermi velocity of Nb ${v}_\mathrm{F}^\mathrm{S}=1.37\times 1{0}^{6}$ m s^−1^, SC gap of Nb *Δ* = 1.2 meV and the mean-free path *l*_mfp_ ≈ 3 nm (refs. ^51,52^).

## Discussion

We now turn to possible scenarios by which SC correlations could be introduced into the edge states through CAR processes, starting first with a scenario in which the SC finger defines a trivial SC region, such that no chiral Majorana edge state can form. The Nb finger is itself trivial and, under certain circumstances, the induced proximitized SC state in the TI surface can also be trivial^23^. Apart from the absence of full spin polarization (Supplementary Notes 9 and 10), this scenario is essentially identical to that of the *ν* = 1 quantum Hall state that has previously been extensively discussed^11–13,17,47,53^. Note, however, that the disordered nature of the QAHI surface would cause the Andreev edge state to become diffusive and results in an equal mix of electrons and holes, such that the Andreev edge state will not contribute to ${R}_{{{{\rm{D}}}}}^{{{{\rm{i}}}}}$. Taking *ξ*_s_ to be the SC coherence length of the mediating superconductor, the CAR processes induce SC correlations across the SC finger and gives rise to negative ${R}_{{{{\rm{D}}}}}^{{{{\rm{i}}}}}$ when the finger width *W*_SC_ is shorter than *ξ*_s_. When *W*_SC_ ≫ *ξ*_s_, the transport along the Andreev edge state dominates over CAR.

**Fig. 4:**
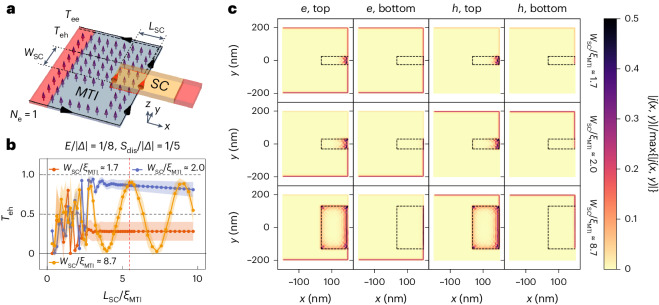
Quantum transport simulation of CAR in a proximitized QAHI thin film. **a**, Schematic of the transport simulation set-up with a magnetic TI (MTI) thin film in the QAHI state. The film is covered by a SC finger over a region with length *L*_SC_ and width *W*_SC_. We consider that the top surface of the MTI below the SC finger has been shifted out of the magnetic gap and proximitized into the TSC regime. The leads (red) are set to be semi-infinite. **b**, The disorder-averaged electron to hole conversion probability *T*_eh_ (standard deviation indicated by shading) across the TSC region at a small bias energy *E* is shown as a function of *L*_SC_ for three selected values of *W*_SC_. **c**, The components of local current densities carried by electrons and holes as well as at the top and bottom surfaces, plotted for the three different widths of the SC finger (indicated by black dashed lines) used in **b**. The finger length under consideration, indicated by the vertical red dashed line in **b**, yields *T*_eh_ > 0.5 for two out of three examples, corresponding to a regime dominated by CAR for a narrow finger and by the chiral Majorana edge-channel interference for a wide finger. Source data

An alternative scenario is that the proximitized TI surface realizes a topological superconducting (TSC) region that hosts a single chiral Majorana edge mode. This can happen, for instance, if the Nb slightly dopes the TI surface to make the chemical potential lie above the magnetic gap and only the top surface is proximitized^2^. In this case, for a wide finger, an incoming electron hitting the TSC region splits into two chiral Majorana modes that take opposite paths enclosing the region covered by the finger. The two chiral Majorana modes recombine on the opposite side of the TSC region as either an electron or a hole, depending, in principle, on the number of residual vortices trapped in the SC region enclosed by the path^54,55^. However, as the chiral Majorana modes have a finite spatial extent and the QAHI surface is disordered, these processes will probably self-average in our several-micrometres-long Nb finger, resulting in an equal mix of electrons and holes transmitted to the opposite side of the finger due to the chiral Majorana modes, such that ${R}_{{{{\rm{D}}}}}^{{{{\rm{i}}}}}\approx 0$. On the other hand, a narrow finger, *W*_SC_ ≲ *ξ*_s_, allows CAR to the opposite edge through the bulk of the proximitized TSC region and leads to ${R}_{{{{\rm{D}}}}}^{{{{\rm{i}}}}} < 0$, as in the previous scenario of trivial SC. We can visualize these qualitatively different regimes of the TSC scenario in quantum transport simulations (Fig. 4a) with a microscopic tight-binding model appropriate for a proximitized QAHI in the TSC regime (see Methods for details). Our simulation results in Fig. 4b,c show that, when the SC finger is much wider than the induced SC coherence length, the current on the top surface is carried by chiral Majorana modes travelling around the proximitized section, with the finger length and the width both affecting the interference. For example, the plot in Fig. 4b for a wide finger shows that the electron to hole conversion probability *T*_eh_ oscillates regularly as a function of the finger length *L*_SC_ when *L*_SC_ ≫ *ξ*_s_. Here, *T*_eh_ > 0.5 means that holes predominantly come out of the finger into the downstream edge due to the interference of the chiral Majorana modes. In a real situation with a long finger, such an oscillating *T*_eh_ would self-average to 0.5, resulting in ${R}_{{{{\rm{D}}}}}^{{{{\rm{i}}}}}\approx 0$. When the finger is narrower (*W*_SC_ ≈ *ξ*_s_), a qualitatively different regime is obtained. In that case, *T*_eh_ is very sensitive to *L*_SC_ for *L*_SC_ ≲ *ξ*_s_, but it stabilizes at large *L*_SC_ to a nearly fixed value that depends sensitively on *W*_SC_. Figure 4b shows the behaviour of *T*_eh_ for two different widths in the narrow regime. These are stabilized at large *L*_SC_ to *T*_eh_ values larger and smaller than 0.5. The simulated local current densities (Fig. 4c) suggest that there are no more well-separated chiral Majorana modes in this regime and that the electron to hole conversion can be attributed to a CAR process that occurs mainly near the QAHI edge.

Therefore, our simulations suggest that, like the trivial SC case, the CAR process can indeed become dominant in the TSC case. We should nevertheless note that the stabilized value of *T*_eh_ for a narrow finger at large *L*_SC_ is strongly dependent on *W*_SC_ in our simulation and is not always larger than 0.5 (Supplementary Note 11), which implies that ${R}_{{{{\rm{D}}}}}^{{{{\rm{i}}}}}$ would fluctuate between negative and positive values as a function of *W*_SC_ in the narrow finger regime. Similar oscillatory behaviour has also been predicted by theoretical calculations for the trivial SC case^11–13^. However, in our experiment, we found Δ*R*_D_ (= ${R}_{{{{\rm{D}}}}}^{{{{\rm{i}}}}}$) to be always negative for narrow fingers, as was also the case with similar experiments on graphene with a trivial SC finger^17,20^. This stability of negative ${R}_{{{{\rm{D}}}}}^{{{{\rm{i}}}}}$ points to the existence of additional physics that are not captured in our simulations. In fact, the reason for the stable dominance of CAR in real experiments is an interesting subject in its own right^13,47,53^. For example, the dissipative channel through vortices in the SC finger, which is not included in our simulations, could be playing a role^19,47^. In this regard, in related experiments to probe the Andreev edge states with a wide SC electrode, oscillatory ${R}_{{{{\rm{D}}}}}^{{{{\rm{i}}}}}$ and stably negative ${R}_{{{{\rm{D}}}}}^{{{{\rm{i}}}}}$ were both reported^18,19^. The latter is surprising^56^, and possible explanations for the dominance of electron to hole conversion in the Andreev edge states have also been proposed^19,46,57,58^. Our result extends the case of the stable dominance of electron to hole conversion and calls for a better theoretical understanding.

One can see from the above considerations that both trivial and non-trivial scenarios are consistent with our observations. Irrespective of its nature, our observation *ξ*_CAR_ ≫ *ξ*_Nb_ implies that CAR occurs through the superconductivity of the proximitized magnetic TI surface, rather than the SC finger itself. This makes sense, since Nb has negligible spin–orbit coupling and the finger on the top surface does not naturally result in processes coupling to the bottom surface, whereas our simulations suggest that the bottom surface needs to be involved in the CAR processes in the QAHI platform. Furthermore, the dependence on the magnetic history of the device suggests that trapped vortices or the magnetic disorder profile play a role, which is natural in the above scenario for CAR through the proximitized surface. If *ξ*_CAR_ is taken as the SC coherence length in the QAHI surface, a simple estimate gives the induced SC gap *Δ*_ind_ ≈ 0.04 meV (Supplementary Note 16).

An obvious next step is to confirm whether the induced 2D superconductivity is topological and is associated with chiral Majorana edge states. A possible experiment to address this question would be based on a device like ours but with a much shorter finger electrode, such that the interference between the two chiral Majorana edge states travelling along either sides of the finger can be detected without self-averaging. A transmitted charge switching between an electron and a hole depending on the number of vortices in the finger would give strong evidence for chiral Majoranas^54,55^. Furthermore, by putting two SC fingers close enough together to make a Josephson junction and by applying a voltage pulse across the junction, one could inject an edge vortex in the chiral Majorana edge state. This edge vortex is a non-abelian zero mode and experiments to confirm its non-abelian nature have been theoretically proposed^24^. Therefore, the platform presented here offers ample opportunities to address topological superconductivity, Majorana physics and non-abelian zero modes.

## Methods

### Material growth and device fabrication

The V-doped (Bi_*x*_Sb_1−*x*_)_2_Te_3_ thin films were grown on InP (111)A substrates by molecular beam epitaxy in a ultrahigh vacuum. High-purity V, Bi, Sb and Te were co-evaporated onto the substrate, which was kept at a temperature of 190 °C to produce a uniform film of thickness ~8 nm. The chemical potential was tuned into the magnetic gap for an optimized Bi:Sb beam-equivalent-pressure ratio of 1:4. A capping layer of 4 nm Al_2_O_3_ was made ex situ with atomic layer deposition at 80 °C using Ultratec Savannah S200 to protect the film from degradation in air. The Hall-bar devices were patterned using standard optical lithography techniques. The narrow Nb/Au SC contacts (45 nm/5 nm) and the Ti/Au normal metal contacts (5 nm/45 nm) were defined using electron-beam lithography. The Al_2_O_3_ capping layer was selectively removed in heated aluminium etchant (Type-D, Transene), before the sputter-deposition of the Ti/Au and Nb/Au layers in a ultrahigh vacuum. Devices A–E reported in this paper were fabricated simultaneously on the same wafer, whereas device F was made on a separate wafer. A clean QAHE without the need for gating was observed in all devices. Scanning electron microscopy was used to determine the width of the Nb electrodes, which were covered with 5-nm-thick Au to avoid oxidation.

### Measurement set-up

The transport measurements were performed at a base temperature of 17–25 mK in a dry dilution refrigerator (Triton 200, Oxford Instruments) equipped with a 8 T SC magnet. All the data presented in Main were measured using a standard d.c. technique with nanovoltmeters (2182A, Keithley) and a current source (2450, Keithley). The a.c. data shown in Supplementary Note 4 were measured using a standard a.c. lock-in technique at low frequency (3–7 Hz) using lock-in amplifiers (LI5640 and LI5645, NF Corporation). The magnetization measurements were performed using a commercial superconducting quantum interference device (SQUID) magnetometer (MPMS3, Quantum Design). The sample was mounted in a plastic straw, self-clamped, with the sample surface perpendicular to the applied magnetic field.

### Quantum transport simulations

We performed the quantum transport simulations using the KWANT^59^ package, by considering a 2 × 4-orbital two-dimensional tight-binding model (on a square lattice with lattice constant *a* = 2 nm), based on the following Bogoliubov–de Gennes model Hamiltonian for a proximitized magnetic TI (MTI) thin film^23,31,60^:

2 $${H}_{{{{\rm{BdG}}}}}\big({k}_{x},{k}_{y}\big)=\left(\begin{array}{rc}{H}_{{{{\rm{MTI}}}}}\big({k}_{x},{k}_{y}\big)-\mu &-\mathrm{i}{\sigma }_{y}\big(1+{\rho }_{z}\big){{\Delta }}/2\\ \mathrm{i}{\sigma }_{y}\big(1+{\rho }_{z}\big){{{\Delta }}}^{* }/2&\mu -{H}_{{{{\rm{MTI}}}}}^{* }\big(-{k}_{x},-{k}_{y}\big)\end{array}\right),$$

3 $${H}_{{{{\rm{MTI}}}}}\big({k}_{x},{k}_{y}\big)=\hslash {v}_{{{{\rm{D}}}}}\big({k}_{y}{\sigma }_{x}-{k}_{x}{\sigma }_{y}\big){\rho }_{z}+\left[{m}_{0}+{m}_{1}\big({k}_{x}^{2}+{k}_{y}^{2}\big)\right]\;{\rho }_{x}+{M}_{z}{\sigma }_{z},$$

with *σ*_*x*,*y*,*z*_ and *ρ*_*x*,*y*,*z*_ the Pauli matrices acting on the spin and pseudospin (for the top and bottom surfaces) degrees of freedom, respectively, and *μ* the chemical potential. We set the Dirac velocity *ℏv*_D_ = 3 eV Å, the top–bottom surface hybridization *m*_0_ = −5 meV and *m*_1_ = 15 meV Å^2^, out-of-plane magnetization strength *M*_*z*_ = 50 meV, and proximity-induced *s*-wave pairing potential *Δ* (on the top surface) with ∣*Δ*∣ = 10 meV (yielding an induced SC coherence length *ξ*_MTI_ = *ℏv*_D_/∣*Δ*∣ = 30 nm). These model parameters yielded a magnetic gap *E*_gap_ = 2(*M*_*z*_ − ∣*m*_0_∣) = 90 meV, meaning that the magnetic gap edge was 45 meV above the Dirac point. We considered the chemical potential *μ* = 25 meV, such that the Fermi level was nearly centred between the Dirac point and the magnetic gap edge. To obtain a TSC regime in the region below the SC finger, we introduced a local shift of Δ*μ* = 75 meV to bring the local Fermi level well above the magnetic gap. Nonmagnetic (for example, electrostatic) disorder was considered by adding a Gaussian random field to the on-site energies of the TI thin film near the position of the SC finger. The disorder was characterized by the disorder strength *S* = 2 meV (the standard deviation of the Gaussian) and spatial correlation length *λ* = 10 nm. Note that the model parameters did not reflect the device properties quantitatively, as that would have required a scattering region several orders of magnitude larger than presently considered in our simulations (in particular, due to the much larger SC finger size and induced SC coherence length). Our aim was to investigate the CAR (*ξ*_MTI_ ≈ *W*_SC_) and Majorana interference (*ξ*_MTI_ ≫ *W*_SC_) regimes qualitatively.

## Online content

Any methods, additional references, Nature Portfolio reporting summaries, source data, extended data, supplementary information, acknowledgements, peer review information; details of author contributions and competing interests; and statements of data and code availability are available at 10.1038/s41567-024-02574-1.

## Supplementary information

Supplementary Information Supplementary Notes 1–16, which include Figs. 1–13.

## Source data

Source Data Fig. 1 Measurement source data.

Source Data Fig. 2 Measurement source data.

Source Data Fig. 3 Measurement source data.

Source Data Fig. 4 Simulation source data.

## Data Availability

Raw data used in the generation of Figs. 1–4 and Supplementary Figs. 1–13 are available via Zenodo at 10.5281/zenodo.11231864 (ref. ^61^). Source data are provided with this paper.

## Code availability

The code used in the quantum transport simulations is available via Zenodo at 10.5281/zenodo.11231864 (ref. ^61^) along with the raw data.
