# Supplementary material for: Induced superconducting correlations in a quantum anomalous Hall insulator
Source: Nat Phys. 2024 Jul 10;20(10):1589–95. doi: 10.1038/s41567-024-02574-1 (PMC11473362; doi:10.1038/s41567-024-02574-1)
Supplement: Supplementary file 1 — Supplementary Notes 1–16, which include Figs. 1–13. [file 41567_2024_2574_MOESM1_ESM.pdf]

nature physics

Supplementary information https://doi.org/10.1038/s41567-024-02574-1

Induced superconducting correlations in a quantum anomalous Hall insulator

In the format provided by the authors and unedited

Supplementary Information for "Induced superconducting correlations in a quantum anomalous Hall insulator"

Supplementary Note 1 Contact Resistance for Ti/Au contact

Figure S1a shows the schematics of the 3-terminal measurement for the contact resistance of contact 1. Figure S1b shows the voltage $V_{6\text{-}1}$ as a function of the DC current $I_{2\text{-}1}$ recorded at 17 mK in different magnetic fields. For an upward, out-of-plane magnetization, the 3-terminal (downstream) resistance $R_{2\text{-}1,6\text{-}1} \equiv V_{6\text{-}1}/I_{2\text{-}1}$ consists only of the sample resistance and the contact resistance. Before the current-induced breakdown of the QAHE, the sample resistance is zero. Hence, the slope of 3.5 Ω in the pre-breakdown regime is the contact resistance of the Ti/Au contact 1.

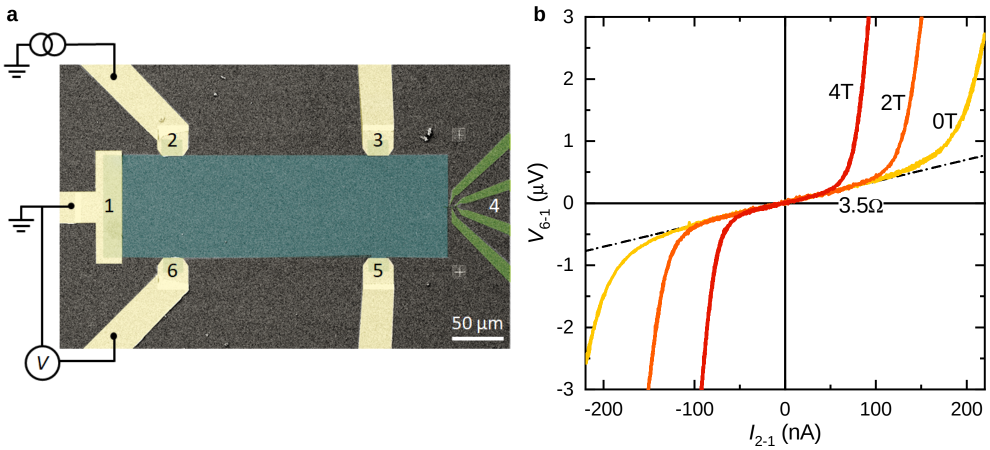

Figure S1: **Three-terminal I-V characteristics in device A for the Ti/Au contact 1. a,** False-colour scanning-electron-microscope image of device A from Fig.1a, including the measurement schematics. The current flew from contact 2 to 1, and the voltage was measured between contacts 6 and 1. For an upward, out-of-plane magnetization ($M > 0$), the chiral 1D edge state propagates in the counter-clockwise direction. **b,** Plots of the 3-terminal voltage $V_{6\text{-}1}$ as a function of the DC current $I_{2\text{-}1}$ for various magnetic field H at 17 mK. The breakdown current of the QAHE decreases with increasing H. The dashed line is a linear fit to the pre-breakdown regime, yielding the slope of ∼3.5 Ω that corresponds to the contact resistance of the Ti/Au contact 1.

Supplementary Note 2 Estimation of the normal-state Nb contribution

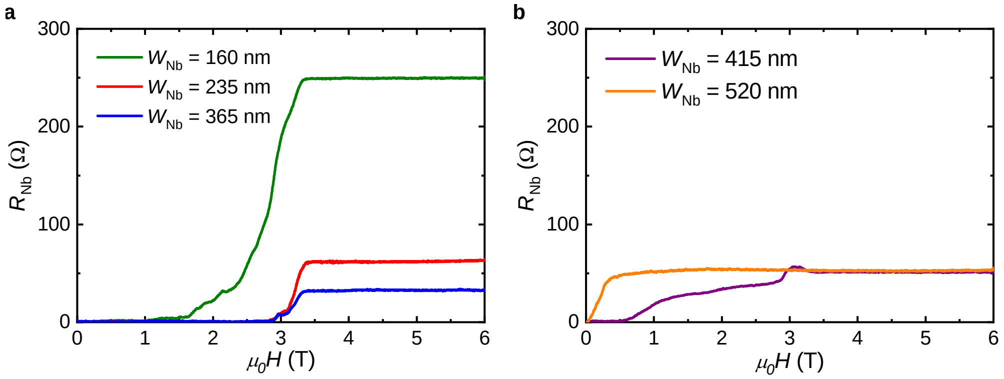

Figure S2: **Magnetic-field dependence of the Nb electrode resistance.** **a,** The 4-terminal Nb resistance R_{Nb} of device A ($W_{\text{Nb}} = 160$ nm), device B ($W_{\text{Nb}} = 235$ nm), and device C ($W_{\text{Nb}} = 365$ nm). **b,** R_{Nb} of device D ($W_{\text{Nb}} = 415$ nm) and device E ($W_{\text{Nb}} = 520$ nm). Although the Nb electrodes on all the devices were fabricated simultaneously, the resistivity of Nb and the upper critical field H_{c2} differ among the devices.

In the main text, Figs. 1a-b show SEM pictures of device A. The Nb electrode contains four contacts (4a, 4b, 4c, and 4d) to allow for a 4-terminal resistance measurement, with separations $L_{\text{Nb,sect.}} \equiv L_{\text{a-b}} = L_{\text{b-c}} = L_{\text{c-d}} = 2.5$ μm. The width is $W_{\text{Nb}} = 160$ nm along the full length of the Nb electrode. The overlap with the V-doped $(\text{Bi}_x\text{Sb}_{1-x})_2\text{Te}_3$ thin film is $L_{\text{Nb,film}} = 5$ μm. The small Nb section on the InP substrate between the edge of the thin film and contact 4a has a length of $L_{\text{Nb,InP}} = 1.2$ μm. Devices B, C, D, and E are identical to device A except for the width of the Nb electrode: $W_{\text{Nb}} = 235$, 365, 415, and 520 nm, respectively. Figure S2 shows the 4-terminal Nb resistance R_{Nb} $(= V_{\text{4b}-\text{4c}}/I_{\text{4a}-\text{4d}})$ as a function of the applied magnetic field. While devices A–E are on the same wafer and the Nb electrodes were fabricated simultaneously, the resistivity of Nb and the upper critical field H_{c2} differ among the devices. The normal-state resistance $R_{\text{Nb,InP}}$ of the $L_{\text{Nb,InP}}$-section contributes to the downstream resistance through $R_{\text{D}} = R_{\text{QAHI}} + R_{\text{Nb,InP}} + R_{\text{contact}} + R^{\text{i}}_{\text{D}}$ as explained in the main text. To estimate $R_{\text{Nb,InP}}$ for each device,

the Nb resistance is rescaled by $L_{\text{Nb,InP}}/L_{\text{Nb,sect.}} = 1.2$ μm / 2.5 μm. This $R_{\text{Nb,InP}}$ was also used in the calculation of the data points for $\Delta R_{\text{D}} = -[R_{\text{D}}(H > H_{\text{c2}}) - R_{\text{D}}(H < H_{\text{c2}}) - R_{\text{Nb,InP}}]$ shown in Fig.3b.

Supplementary Note 3 *I*-*V* characteristics at different magnetic fields in device A

In the main text, Fig. 1e shows the magnetic-field dependence of R_{D}; the blue symbols represent the slopes at I_{DC} = 0 extracted from the I-V curves shown in Fig. S3. Negative R_{D} is observed for $\mu_0 H < 1$ T. Moreover, notice the large noise amplitude for the V_{D}-vs-I_{DC} curves displaying negative slopes, whereas a lower noise level is observed for curves measured above the H_{c2} of Nb. This indicates that the noise is intrinsic to the CAR process in this system.

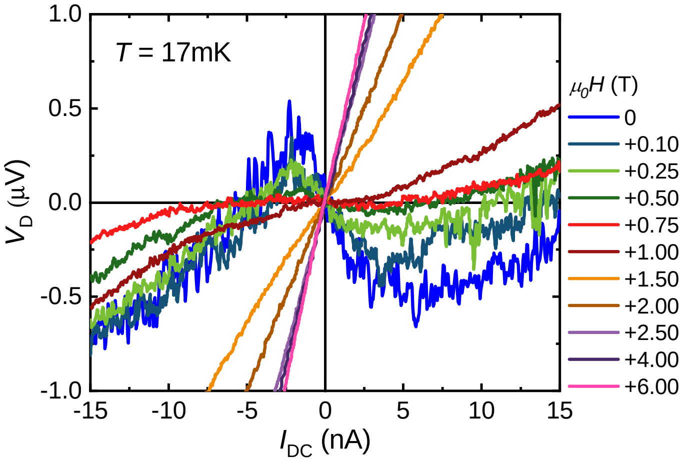

Figure S3: ***I*-*V* characteristics at different magnetic fields in device A.** The negative slope in the zero-current limit, confirming the negative R_{D}, is reproducibly observed below 1 T, where the Nb electrode is still superconducting.

Supplementary Note 4 Comparison of R_D measured with DC and AC techniques

The plots of V_D vs I_{DC} shown in Fig. S3 were obtained with a DC technique. Up to a DC current of $|I_{DC}| \lesssim 3$ nA, the slope of V_D (and hence R_D) is negative below 1 T. To verify the negative R_D, the sample is remeasured with an AC lock-in technique with a small AC excitation current of $I_{RMS} = 1$ nA (i.e. $I_{peak} = 1.41$ nA) for the same experimental setup as shown in Fig. 1a. Figure S4 shows that the result of the AC measurement agrees well with the slopes of the I-V curves at I_{DC} = 0 measured with the DC technique. Hence, the negative R_D in device A is reproducible between the AC and DC techniques. We note that the sharp spike in R_D near zero magnetic field seen in the AC-measurement data in Fig. S4 is an artifact most likely related to a sharp increase in the temperature upon crossing $H = 0$ due to a magnetocaloric effect, similar to observations by other research groups [1–3].

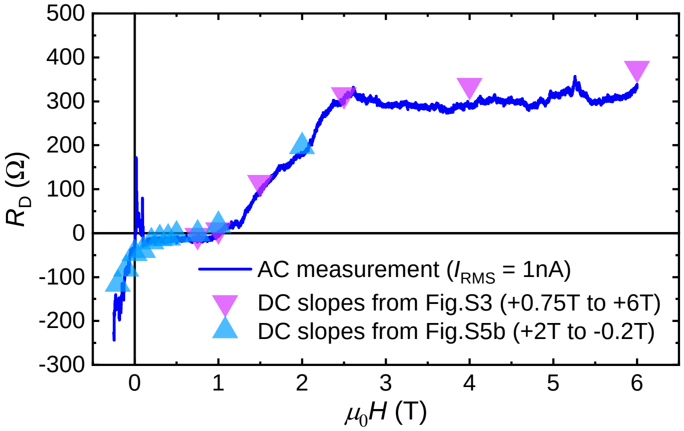

Figure S4: **Validation of the R_D values in device A.** The solid blue line shows R_D measured continuously with an AC technique with $I_{RMS} = 1$ nA at 25 mK as a function of the applied magnetic field; this R_D result is consistent with the R_D values extracted from the slopes of the I-V curves at I_{DC} = 0 measured with the DC technique (magenta and cyan symbols).

Supplementary Note 5 Effect of the magnetic-field-sweep history on R_D in device A

After taking the data shown in Fig. S3 (for which H was increased from 0 to 6 T), we reduced H back to 0 T and took the I-V data shown in Fig. S5a in the order of 0 , -0.1, and -0.25 T. Then, we increased the magnetic field to 2 T and decreased it to -0.2 T, during which we took the series of I-V curves at different magnetic fields shown in Fig. S5b. Interestingly, the R_D values in the zero-current limit obtained for the series shown in Fig. S5b are essentially consistent with those obtained in the series shown in Fig. S5a, see Fig. S5d. This suggests that the system has metastable disorder profiles, and it remained in the same profile between the measurements of Fig. S5a and Fig. S5b, while the profile changed from that in the measurements of Fig. S3 (i.e. Fig. 1e in the main text). The temperature dependence data shown in Fig. 2b of the main text were measured after we took the data in Fig. S5b and set the magnetic field to zero again. To check for the effect of thermal cycling, we measured the 0-T I-V curves at 17 mT before and after the sample was heated to 200 mK, and the result is shown in Fig. S5c. It appears that the thermal cycling has little effect on R_D.

To summarize the effect of the magnetic-field-sweep history in device A, Fig. S5d shows the R_D values obtained in four different magnetic-field sweeps performed to take the data shown in Figs. S3, S5a, S5b, and S5c. Altogether, the obtained negative slopes of -210 Ω (Fig. 1f), -215 Ω (Fig. S5a), -117 Ω (Fig. S5b), -92 Ω (Fig. S5c), and -72 Ω (Fig. S5c) are used as the values of $R_\mathrm{D}(H < H_\mathrm{c2})$ for each magnetic cycle to calculate the five data points of $\Delta R_\mathrm{D} = -[R_\mathrm{D}(H > H_\mathrm{c2}) - R_\mathrm{D}(H < H_\mathrm{c2}) - R_\mathrm{Nb,InP}]$ shown in Fig. 3b of the main text for devicd A, along with $R_\mathrm{D}(H > H_\mathrm{c2}) = 310\,\Omega$ (obtained from the data in Fig. S3) and $R_\mathrm{Nb,InP} = 120\,\Omega$ (Fig. S2).

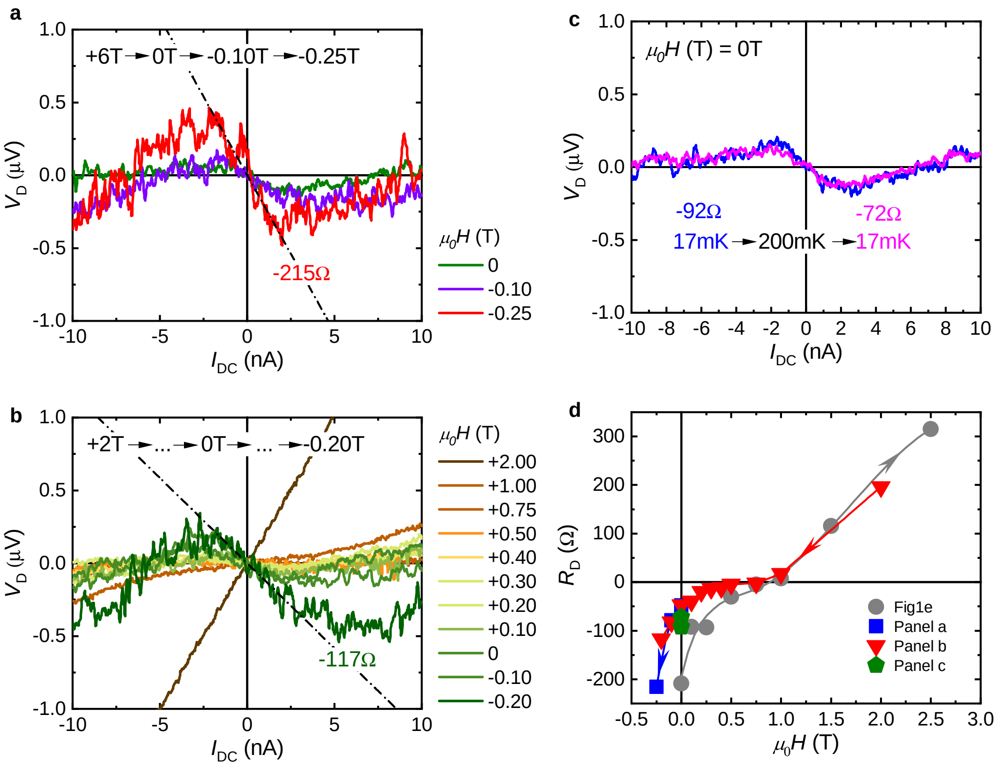

Figure S5: **Effect of the magnetic-field-sweep history observed in device A.** **a,** I-V characteristics measured at 17 mK in the order of 0 , -0.1, and -0.25 T directly after taking the magnetic-field-dependence data shown in Fig. S3. **b,** I-V characteristics measured at 17 mK in decreasing magnetic fields from 2 T to -0.2 T directly after taking the data shown in panel **a** and bringing the magnetic field to 2 T. The dashed lines in panel **a** and **b** show the maximum negative slopes observed at -0.25 T and -0.2 T, respectively. **c,** I-V characteristics measured at 17 mK in 0 T before (blue) and after (magenta) thermal cycling to 200 mK, directly after taking the data shown in panel **b**. **d,** Collection of the R_D values obtained in four different magnetic-field sweeps. Blue, red, and green symbols are the slopes at I_{DC} = 0 extracted from the data in panels **a**, **b**, and **c**, respectively. The gray symbols are the discrete data points up to 2.5 T shown in Fig. 1e.

Supplementary Note 6   Current- and temperature-induced breakdown of the QAHE

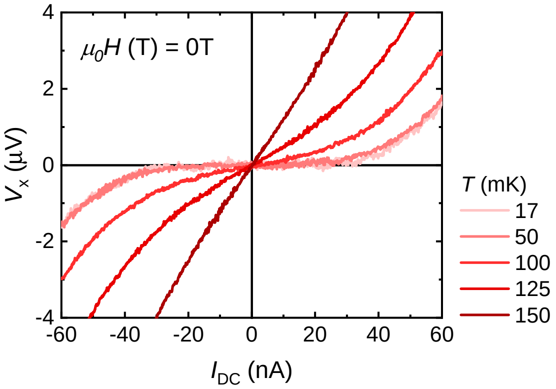

Figure S6: **Breakdown of the QAHE.** Plots of the 4-terminal longitudinal voltage V_x vs I_{DC} in 0 T ($M > 0$) measured in device A at various temperatures. The thermal activation of charge carriers with increasing temperature gives rise to a parallel dissipative conduction channel, causing the zero-resistance state of the QAHI to disappear at $\sim$100 mK, while the current-induced breakdown of the QAHE causes a finite V_x above $\sim$30 nA at 17 and 50 mK.

In Fig. 2b of the main text, the 4-terminal longitudinal resistance R_{xx} is shown as a function of temperature. The data points of R_{xx} were extracted from the I-V curves shown in Fig. S6 as the slope at I_{DC} = 0; here, the current was set to flow between contacts 1 and 4d, and the voltage between contacts 6 and 5 was measured. The 17-mK and 50-mK curves present a well extended zero-voltage plateau up to $\sim$30 nA, after which the current-induced breakdown of the QAHE occurs. At higher temperatures, the zero-resistance state is not realized due to the thermal activation of charge carriers into the gapped 2D surface states of the QAHI [1,4–6].

Supplementary Note 7   Downstream resistance measured with wider Nb electrodes

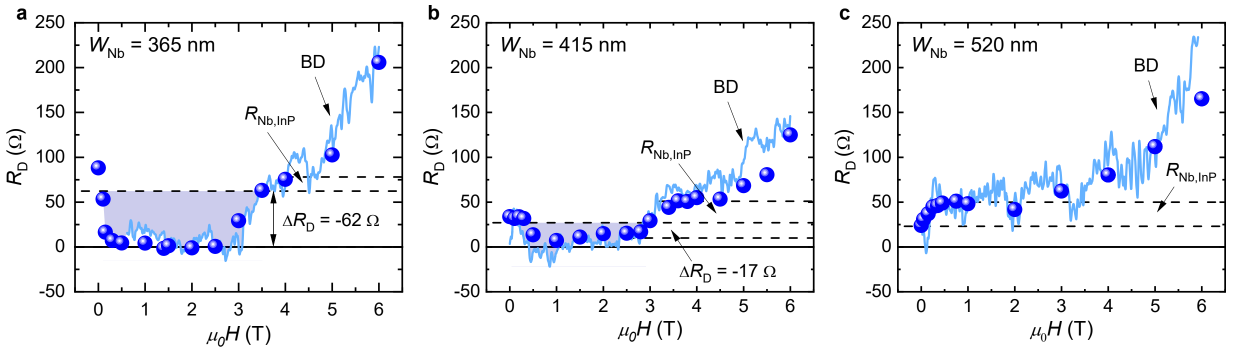

Figure S7: **Downstream resistance in devices with a wider Nb electrode. a-c,** Light blue lines show the magnetic-field dependencies of R_D at 25 mK measured with $I_{DC} = 2$ nA in device C (**a**, $W_{Nb} = 365$ nm), device D (**b**, $W_{Nb} = 415$ nm) and device E (**c**, $W_{Nb} = 520$ nm). Blue symbols represent the slopes in the I-V curves at I_{DC} = 0. Note that above ~4.5 T, the breakdown (BD) of the QAHE starts to dominate R_D in all these devices. The distance between two dashed lines in each panel mark the estimated normal-state Nb contribution $R_{Nb,InP}$ pointed by an arrow, based on which the CAR contribution ΔR_D is estimated.

Figure S7 shows R_D as a function of the applied magnetic field measured at 25 mK in devices C, D, and E having the Nb-electrode width of 365, 415, and 520 nm, respectively. Note that the increase in R_D above $\sim$4.5 T observed in all devices is due to the breakdown of the QAHE, see Fig. 1d of the main text. In devices C and D, the normal-state Nb contribution was $\sim$16 Ω and $\sim$24 Ω, yielding ΔR_D of about -62 Ω and -17 Ω, respectively, when one compares R_D before and after the superconductivity is suppressed. In device E, on the other hand, the normal-state Nb contribution of $\sim$27 Ω accounts for the full increase in R_D upon the suppression of superconductivity. Hence, ΔR_D is zero for the 520-nm-wide Nb electrode of device E. The ΔR_D values of these devices are included in Fig. 3b of the main text.

Supplementary Note 8 Landauer-Büttiker formalism

For the measurement configuration shown in Fig. 1a of the main text, the current flows from contact 1 to 4. The chiral edge state runs counter-clockwise along the sample edge for an upward, out-of-plane magnetization. The current-voltage relation in the linear-response Landauer-Büttiker formalism [7,8] is given by

$$I_i = \sum_{j=1,j\neq 4}^{6} a_{ij}\left(V_j - V\right), \tag{S1}$$

where I_i is the current flowing into contact i, V_j is the potential at contact j, and V is the potential of the superconducting electrode (contact 4). The proportionality coefficients $a_\mathrm{i\,j}$ in Eq. S1 at zero temperature are given by

$$a_{ij} = \frac{e^2}{h}\left(N_i^\mathrm{e}\delta_{ij} - T_{ij}^\mathrm{ee} + T_{ij}^\mathrm{eh}\right). \tag{S2}$$

Here, N_i^e is the number of available channels for electron-like excitation in contact i:

$$N_i^\mathrm{e} = \sum_{j=1,j\neq 4}^{6}\left(T_{ij}^\mathrm{ee} + T_{ij}^\mathrm{eh}\right), \tag{S3}$$

where $T_\mathrm{ij}^\mathrm{ee}$ ($T_\mathrm{ij}^\mathrm{eh}$) is the transmission probability of an electron from the j-th contact to arrive as an electron (hole) at the i-th contact. Since a QAHI possesses only a single chiral edge state, $N_i^\mathrm{e} = 1$ for all contacts. Notice that the potential difference in Eq. S1 is expressed with respect to the potential of the grounded superconducting contact 4 ($V = V_4 = 0$), and the summation in Eqs. S1 and S3 runs only over the normal metal contacts. The non-zero transmission probabilities T_ij are simply T_{12}^ee = T_{23}^ee = T_{56}^ee = T_{61}^ee = 1, T_{35}^ee, and T_{35}^eh. The non-zero proportionality coefficients $a_\mathrm{i\,j}$ then

become

$$a_{11} = a_{22} = a_{33} = a_{55} = a_{66} = \frac{e^2}{h}, \tag{S4}$$

$$a_{12} = a_{23} = a_{56} = a_{61} = -\frac{e^2}{h}, \tag{S5}$$

$$a_{35} = \frac{e^2}{h}\left(-T_{35}^{\mathrm{ee}} + T_{35}^{\mathrm{eh}}\right). \tag{S6}$$

Using $I_1 = -I_4 = I$ and $I_2 = I_3 = I_5 = I_6 = 0$, Eq. S1 gives a set of equations which can be solved for I and V_i. The expressions for the current I, downstream resistance $R_{\mathrm{D}}^{\mathrm{i}}$, and upstream resistance $R_{\mathrm{U}}^{\mathrm{i}}$ for an ideal (dissipationless) superconducting contact [9,10] then become

$$I = \frac{e^2}{h}\left(1 - T_{35}^{\mathrm{ee}} + T_{35}^{\mathrm{eh}}\right) V_{\mathrm{SD}}, \tag{S7}$$

$$R_{\mathrm{D}}^{\mathrm{i}} = \frac{V_{\mathrm{D}}}{I} = \frac{h}{e^2}\left(\frac{T_{35}^{\mathrm{ee}} - T_{35}^{\mathrm{eh}}}{1 - T_{35}^{\mathrm{ee}} + T_{35}^{\mathrm{eh}}}\right), \tag{S8}$$

$$R_{\mathrm{U}}^{\mathrm{i}} = \frac{V_{\mathrm{U}}}{I} = \frac{h}{e^2}\left(\frac{1}{1 - T_{35}^{\mathrm{ee}} + T_{35}^{\mathrm{eh}}}\right), \tag{S9}$$

where $V_{\mathrm{SD}} \equiv V_1 - V_4$, $V_{\mathrm{D}} \equiv V_3 - V_4$, and $V_{\mathrm{U}} \equiv V_5 - V_4$. Notice that $R_{\mathrm{U}}^{\mathrm{i}} - R_{\mathrm{D}}^{\mathrm{i}} = h/e^2$ as expected.

T_{35}^{ee} and T_{35}^{eh} are not independent parameters, they represent transmission probabilities of an electron leaving contact 5 and should satisfy the following relation:

$$T_{35}^{\mathrm{ee}} + T_{35}^{\mathrm{eh}} + T^{\mathrm{D}} = 1, \tag{S10}$$

where T^{D} is the probability of the direct transfer of the electron into the SC contact 4. The $T^{\mathrm{D}} = 0$ condition represents the case of a perfect superconductor, for which an electron with the energy smaller then the SC gap cannot enter SC contact directly, but only through Andreev processes with finite T_{35}^{eh}. In the extreme case of 100% Andreev process with T_{35}^{eh} = 1, one should observe a doubling of the current I (see Eq. S7), resulting in the maximally negative downstream resistance

$-h/(2e^2)$ (see Eq. S8). On the other hand, $T^{\mathrm{D}} = 1$ represents the case when the contact 4 acts as a perfect metal, which can be achieved in our experiment, for example, by applying a magnetic field and fully suppressing the superconductivity in the finger. In this case, $R_{\mathrm{D}}^{\mathrm{i}} = 0$ (see Eq. S8) as expected for an ideal metallic contact. For $0 < T^{\mathrm{D}} < 1$, the observation of a negative downstream resistance $R_{\mathrm{D}}^{\mathrm{i}} < 0$ is a direct indication that $T_{35}^{\mathrm{ee}} < T_{35}^{\mathrm{eh}}$ (see Eq. S8), i.e., there are more holes than electrons that arrive at contact 3.

Both T_{35}^{ee} and T_{35}^{eh} represent total probabilities for electrons and holes to get into the downstream channel after interacting with the SC finger and finally reach the contact 3. It is useful to distinguish between different contributions. In particular, crossed Andreev reflections (CAR) and direct tunneling of electrons from upstream to downstream channel (so called electron co-tunneling, CT) are expected to decay exponentially with increasing width of the finger. We can write $T_{35}^{\mathrm{ee}} = T^{\mathrm{CT}} + T^{\mathrm{N}}$ and $T_{35}^{\mathrm{eh}} = T^{\mathrm{CAR}} + T^{\mathrm{A}}$, where T^{N} and T^{A} represent the probabilities of all other processes at the finger to get into the downstream channel as an electron and hole, respectively. In most processes such as the transport through the Andreev edge state or the chiral Majorana edge state, one would expect an equal mixture of electron and hole on a long finger, i.e. $T^{\mathrm{N}} = T^{\mathrm{A}}$. Moreover, in real devices the SC-QAHI interface is never ideal and always contains a finite contact resistance R_{contact}. The expression for the apparent R_{D} is then given by

$$R_{\mathrm{D}} = R_{\mathrm{D}}^{\mathrm{i}} + R_{\mathrm{contact}} = \frac{h}{e^2} \frac{T^{\mathrm{CT}} - T^{\mathrm{CAR}}}{1 - (T^{\mathrm{CT}} - T^{\mathrm{CAR}})} + R_{\mathrm{contact}}. \tag{S11}$$

This R_{D} becomes negative only when CAR occurs more often than CT and the resulting negative contribution is large enough to overcome R_{contact}. Nevertheless, even when the apparent R_{D} remains positive, one can identify negative $R_{\mathrm{D}}^{\mathrm{i}}$ by subtacting R_{contact} from the apparent R_{D}, which is

done by calclulating ΔR_{D} used in the main text.

Note that in the experimental setup shown in Fig. 1a, there are two additional contributions to the downstream resistance (see Eq. 1): the resistance R_{QAHI} of the QAHI film (which is zero for low probe currents below the breakdown) and the resistance $R_{\mathrm{Nb,InP}}$ of the Nb section lying on the InP wafer between the film edge and the SC contact 4a (which is zero when the Nb is superconducting).

Supplementary Note 9 Wavefunction of chiral edge state

Following similar derivations in Refs. 11, 12, without loss of generality and neglecting coupling to the two split-off bands far from the Fermi-level that do not have a band inversion resulting from the magnetization, we write the two lowest energy states as $|+\uparrow_z\rangle = (|t\uparrow_z\rangle + |b\uparrow_z\rangle)/\sqrt{2}$ and $|-\downarrow_z\rangle = (|t\downarrow_z\rangle - |b\downarrow_z\rangle)/\sqrt{2}$, where $(+)$ is a symmetric (bonding) and $(-)$ is an antisymmetric (anti-bonding) state spread over the top and bottom surface with spin $\uparrow_z$ and $\downarrow_z$ along the magnetization axis. In the basis $(|+\uparrow_z\rangle, |-\downarrow_z\rangle)$ the Hamiltonian of the lowest energy states is then given by

$$H = \begin{pmatrix} m_k - M & -iv(k_x + ik_y) \\ iv(k_x - ik_y) & -m_k + M \end{pmatrix} = (m_k - M)\,\tau_z + v\,(k_y \tau_x + k_x \tau_y)\,, \tag{S12}$$

where $m_k = m + B(k_x^2 + k_y^2) > 0$. A magnetization $M > m$ ensures that there is a band inversion that results in the existence of the chiral edge mode.

We consider an edge state on a boundary parallel to the x-axis such that the state lives in the region $y > 0$ and k_x remains a good quantum number. For $k_x = 0$ we make the Ansatz that the

edge state can be expressed $\psi(y, k_x = 0) = A\boldsymbol{\xi}\exp(-y/\lambda)$, which means that $1/\lambda$ has to satisfy

$$\left[\left(m - M - \frac{B}{\lambda^2}\right)\tau_z + \frac{iv}{\lambda}\tau_x\right]\boldsymbol{\xi} = 0, \tag{S13}$$

which has non-trivial solutions if $\boldsymbol{\xi} = (1, \chi i)/\sqrt{2}$ with $\chi = \pm 1$ and

$$\frac{1}{\lambda} = \frac{-\chi v \pm \sqrt{v^2 + 4B(m - M)}}{2B}. \tag{S14}$$

Since physical states must decay and we consider the case where the edge state is in the region $y > 0$, only $\lambda > 0$ is a valid solution. Furthermore, since $m < M$, only $\chi = -1$ ensures that both spinor components always satisfy this condition. Therefore, the edge state takes the form[11,13]:

$$\psi(y, k_x = 0) = f(y)(|t\uparrow_z\rangle + i\,|t\downarrow_z\rangle) + (|b\uparrow_z\rangle - i\,|b\downarrow_z\rangle))/\sqrt{2} = f(y)(|t\uparrow\rangle + |b\downarrow\rangle), \tag{S15}$$

where $f(y) \sim \exp(-y/\lambda)$ and $\uparrow, \downarrow$ refers to spin in the plane of the QAHI perpendicular to the edge (here, y-direction).

Supplementary Note 10 Difference from the $\nu = 1$ state of a quantum Hall insulator

At first sight, the chiral edge state of a QAHI appears similar to the $\nu = 1$ state of a quantum Hall insulator[14,15]. However, the spin-polarised nature of the $\nu = 1$ state necessitates, for instance, a superconductor with strong spin-orbit coupling or a nonuniform magnetic field distribution in order for CAR processes to occur[9,14,15]. In contrast, the edge state of the QAHI considered in our work is a superposition of spin states on the top and bottom surfaces. Furthermore, when brought into proximity with a superconductor, the resultant doping of the TI surface[16,17] will lead to an induced superconductivity that inherently has strong spin-orbit coupling. In a simple approximation and at

zero-momentum, the wavefunction of the chiral edge in the x-direction of a QAHI takes the form of Eq. S15. If we consider an asymmetry χ between the top and bottum surfaces, the wavefunction is generalized to

$$\Psi(y) = f(y)\left(|t\uparrow\rangle + \chi\,|b\downarrow\rangle\right). \tag{S16}$$

In the isotropic case, $\chi = 1$, the edge state has no net spin-polarisation and the CAR process will not be hindered as long as the SC finger is narrow enough. Superconducting pairing across the finger will be only slightly suppressed by a partial spin-polarisation of the edge states, which may arise in realistic situations [13]. Even in the extreme case of a fully spin-polarised edge, $\chi = 0$, CAR can still occur due to spin-orbit coupling, if the superconductivity is induced on the TI surface.

Supplementary Note 11 Quantum transport simulations

Here, we present more quantum transport simulation results for the setup of Fig. 4a. In Fig. S8, we show T_{ee} and T_{eh} as a function of the bias energy E and the disorder strength S_{dis} for fixed L_{SC} = 191 nm and W_{SC} = 260 nm in the case of no doping, i.e., without shifting the chemical potential outside of the magnetic gap into the TSC regime below the SC finger; the local current density distributions for two representative disorder levels are also shown. In this case, the top surface remains undoped and the chiral edge channel displays perfect CT at low bias and low disorder strengths. When S_{dis} becomes large enough to push the system locally out of the magnetic gap, electron-hole conversion starts to appear ($T_{\mathrm{eh}} > 0$). Note that S_{dis} is the standard deviation of the Gaussian random field added to the on-site energies to simulate the disorder potential. At larger S_{dis}, the disordered region is effectively doped and T_{eh} fluctuates around 0.5 with a large standard

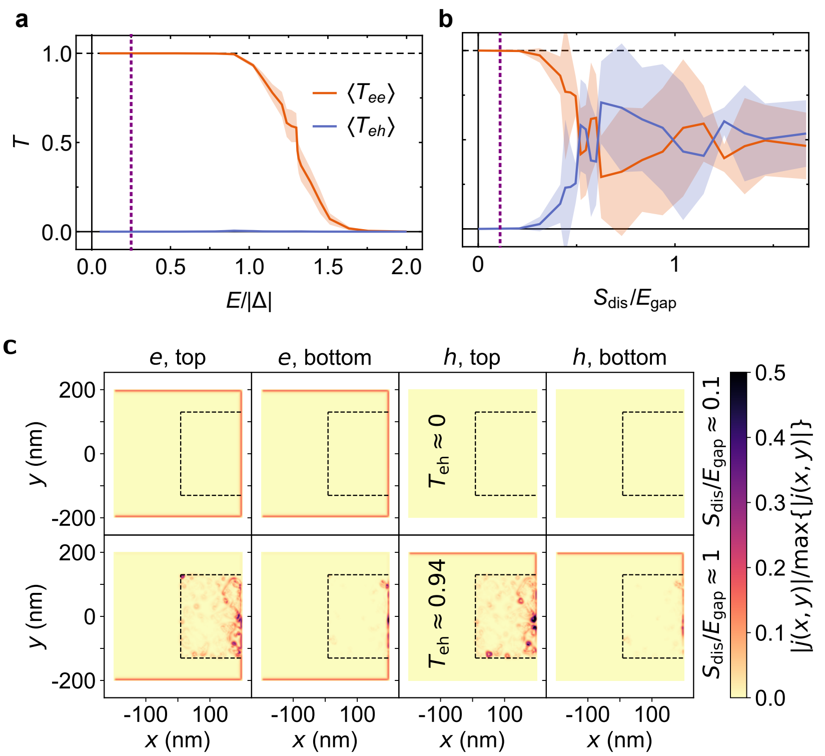

Figure S8: **Quantum transport simulation of proximitized QAHI without doping from the SC finger. a,b,** $\langle T_{ee} \rangle$ and $\langle T_{eh} \rangle$ obtained after averaging the results for various disorder distributions (one standard deviation is indicated by shading) shown as a function of bias energy E ($|\Delta|$ = 10 meV) in **a**, and as a function of the disorder strength S_{dis} (relative to the magnetic gap E_{gap} = 90 meV) in **b**. The disorder strength (bias energy) considered in the calculations for **a** (**b**) is indicated by the dashed purple line in **b** (**a**). Here, we fixed L_{SC} = 191 nm and W_{SC} = 260 nm. **c,** Components of the local current densities for $S_{\text{dis}}/E_{\text{gap}} = \frac{1}{9}$ (top) and $S_{\text{dis}}/E_{\text{gap}} = \frac{10}{9}$ (bottom) at the bias energy $E/|\Delta| = \frac{1}{8}$; for the latter, a disorder configuration that gave a particularly high T_{eh} is chosen for demonstration purpose.

deviation. This scenario is unlikely to apply to the experimental setup, as the QAHI state is well established in the sample. This suggests that a TSC phase due to uniform doping below the SC finger is needed in the proximitized top surface in order to mediate Andreev processes of the chiral edge channel with its peculiar spin polarization [see Eq. (S15)]. Note that, with this simulation setup, we do not consider alternative (trivial) scenarios, e.g., the possibility of the QAHI edge state leaking into a subgap state in the SC finger and undergoing Andreev processes there before

going back into the QAHI edge state as a hole. Furthermore, note that we model the SC lead (shown in Fig. 4a) by a two-dimensional tight-binding model that is lattice-matched to the TI thin film model, considering parameters for a free electron gas with s-wave pairing (Δ). It is only relevant for energies above the SC gap $|\Delta|$ when QP tunneling into the SC lead is possible, yielding $T_{\mathrm{ee}} + T_{\mathrm{eh}} < 1$ (see Fig. S8a). However, in the presence of vortices, tunneling into the SC lead is possible even for $E < |\Delta|$, causing $T_{\mathrm{ee}} + T_{\mathrm{eh}} < 1$ even at low energies.

In Fig. S9, we present T_{eh} as a function of the SC finger length L_{SC} as in Fig. 4b, but for different biases and disorder strengths. Increasing the disorder strength reduces the amplitude of the Majorana edge channel interference pattern around the average $T_{\mathrm{eh}} \leq 0.5$, whereas increasing the bias energy pushes down the amplitude of the interference pattern towards $T_{\mathrm{eh}} = 0$. This indicates that, for obtaining a clean Majorana edge-channel interference pattern in wide fingers and for identifying a qualitatively different CAR/CT-dominated regime in narrow fingers, disorder and bias should be sufficiently small compared to the magnetic gap and the proximity-induced pairing potential.

Although our quantum transport simulations for narrow SC fingers indeed support the possibility of CAR to take place in the QAHI edge, they do not yield a regime with $T_{\mathrm{eh}} > 0.5$ that is robust against small variations in the setup (e.g., finger width or bias energy). This is different from experiment. As we mentioned in the main text, there should be additional physics which causes the stable dominance of CAR in real situations. One such possibility is the dissipation into subgap states in the SC finger (not included in our simulation setup, where $T_{\mathrm{ee}} + T_{\mathrm{eh}} = 1$ is as-

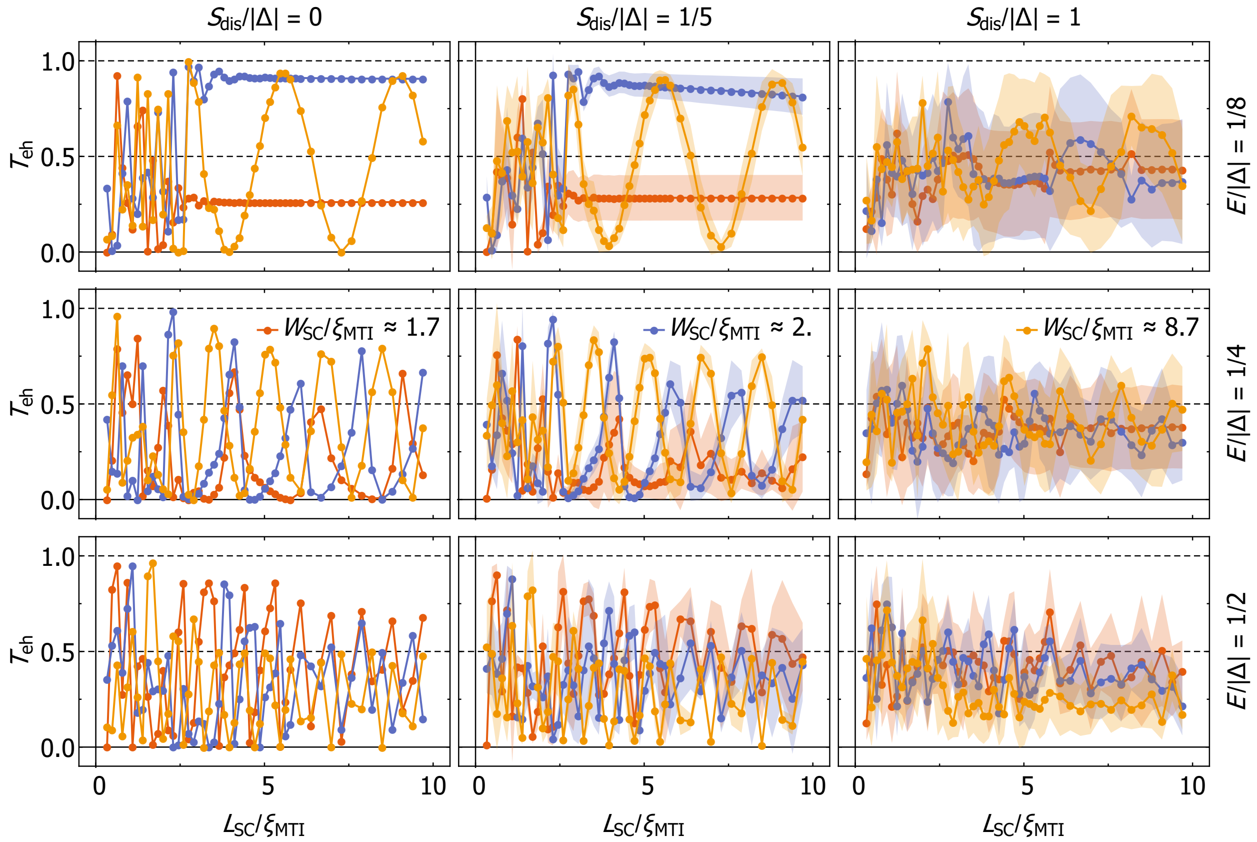

Figure S9: **Quantum transport simulation of crossed Andreev reflection in a proximitized QAHI (extended).** The disorder-averaged electron-hole conversion probability T_{eh} (standard deviation indicated by shading) as a function of SC finger length as in Fig. 4b for different energies E and disorder strengths S_{dis} for three selected SC finger widths (orange, blue, and dark-yellow colour correspond to $W_{\mathrm{SC}}/\xi_{\mathrm{MTI}}$ values of 1.7, 2.0, and 8.7, respectively).

sumed for all subgap energies $E < |\Delta|$), as suggested in Ref. 10. When tunneling of electrons into the SC (which leads to dissipation) is allowed at low energies in addition to the tunneling into the downstream edge, the CT process would compete more with such a tunneling process than CAR, yielding $\langle T_{\mathrm{eh}} \rangle > \langle T_{\mathrm{ee}} \rangle$ even when CAR and CT are equally likely in the case without dissipation. We can further speculate that such an (imbalanced) dissipative process only starts to appear when the SC finger is narrow enough, because for wide fingers the chiral Majorana edge channels are well formed and they may short-circuit the tunneling processes.

We note that our simulations considered the TSC state with a single Majorana mode per edge ($\mathcal{N}$ = 1). Theoretically, there can also be a different TSC state with double Majorana modes per edge ($\mathcal{N}$ = 2) [18]. If a TSC state with $\mathcal{N}$ = 2 is realized on the undoped (or only slightly doped) QAHI surface as considered in Ref. 18, the $\mathcal{N}$ = 2 edge state is equivalent to a single chiral QAH edge state and it provides a direct path for an incoming edge electron to travel to the downstream as argued in Ref. 18, leading to a large positive ΔR_{D} especially for wide fingers where CAR is suppressed. We never observed such a positive ΔR_{D} for wide fingers, and therefore we believe that this scenario is not likely. The other possibility to have a TSC state with $\mathcal{N}$ = 2 is that both top and bottom surfaces are sufficiently doped and each hosts a TSC state with $\mathcal{N}$ = 1, such that the total $\mathcal{N}$ number becomes 2. In this case, the edge state will surround the finger and the transport of electrons and holes for a long and wide finger will self-average to give zero contribution to ΔR_{D}, similar to the case of the Andreev edge state of a trivial SC phase.

Supplementary Note 12   Comparison of the up- and downstream resistances

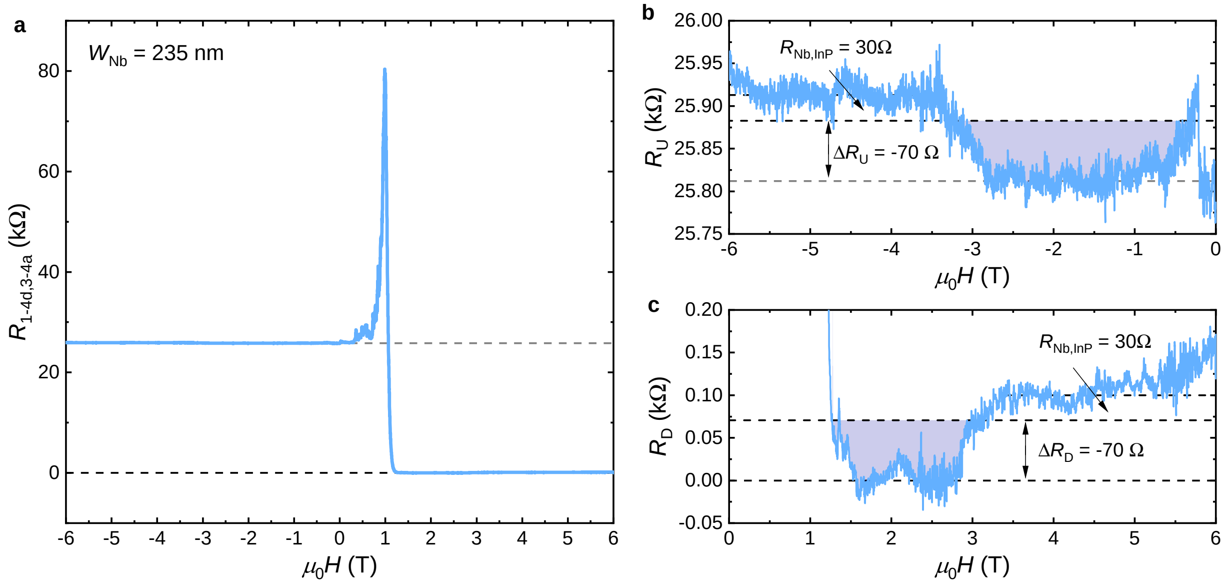

Figure S10: **Magnetic-field dependence of the up- and downstream resistances for the 235-nm-wide Nb electrode of device B. a,** The three-terminal resistance $R_{\text{1-4d,3-4a}}$ changes by h/e^2 when crossing the coercive field upon up-sweep. **b,** Zoom of $R_{\text{1-4d,3-4a}}$ for negative field values where it corresponds to R_U. **c,** Zoom of $R_{\text{1-4d,3-4a}}$ for positive field values where it corresponds to R_D. The relation $R_\text{U} - R_\text{D} = h/e^2$ holds in both the SC and the normal states.

In this work, the focus was made on the measurements of R_D, because a negative R_D presents a clear signature of the CAR process. However, according to Eqs. S8-S9, R_U should change in the corresponding manner so that $R_\text{U} - R_\text{D}$ always yields the quantized value h/e^2. Figure S10 shows $R_{\text{1-4d,3-4a}}$ upon sweeping the magnetic field from -6 T to $+6$ T. For the negative field range up to the coercive field of the QAHI thin film, $R_{\text{1-4d,3-4a}}$ corresponds to R_U, whereas beyond the coercive field $R_{\text{1-4d,3-4a}}$ corresponds to R_D. One can see that $\Delta R_\text{U} = \Delta R_\text{D} = -70\ \Omega$ and the relation $R_\text{U}(-H) - R_\text{D}(+H) = h/e^2$ indeed holds in both the SC and the normal states.

Supplementary Note 13 Effect of sample magnetization on the Nb superconductivity

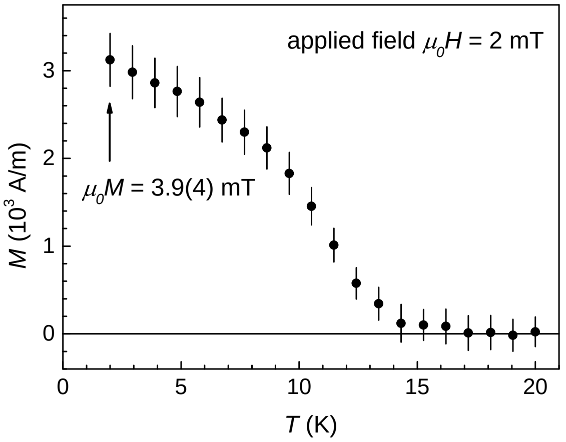

Figure S11: **Magnetization measured using SQUID magnetometry.** Magnetization (M) between 2 and 20 K, after applying at 2 K a saturating field ($\mu_0 H$) of 7 T and reducing it to 2 mT, i.e. to a near-remanence state, and measuring the magnetization upon heating keeping the applied 2 mT field. A non-zero positive field was applied to avoid that trapped-field effects associated with the superconducting magnet could result in an effectively negative applied magnetic field. The error bars are determined based on conventional error propagation, taking into account (i) the standard deviation of multiple SQUID magnetometry measurements at each constant temperature step, (ii) the subtraction of the diamagnetic contribution from the substrate, (iii) the normalization of the magnetic moment with respect to the film volume.

The vicinity to a ferromagnetic film may be detrimental to the superconducting properties of the Nb electrode, due to the magnetic field originating from the film. In order to evaluate if such effects could play a role in our experiments, we carried out magnetization measurements of our QAHI samples using SQUID magnetometry (Quantum Design MPMS®3). The typical magnetization of our samples was found to be about 3×10^3 A/m at 2 K, corresponding to a magnetic induction of about 4 mT. This is well below the lower critical field of Nb, which is $H_{c1} \approx 180$ mT [19]. As an example, Fig. S11 shows the near-remanence magnetization as a function of temperature, measured from 2 to 20 K. Given the square hysteresis of our QAHI films (at 2 K and below), the near-remanent magnetization measured here is approximately equal to both the

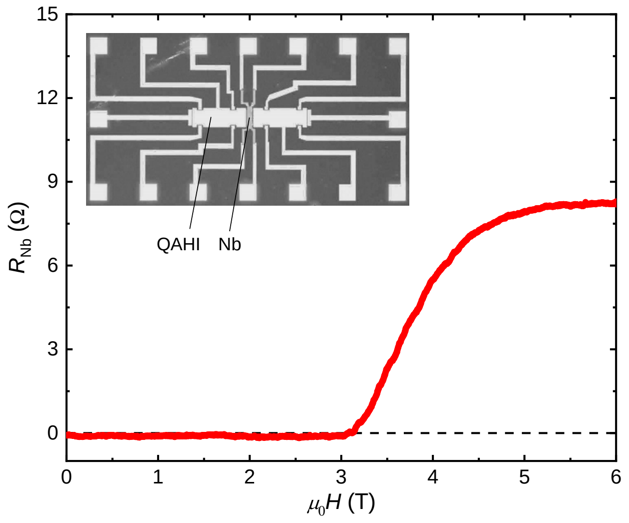

Figure S12: **Magnetic-field dependence of the resistance of a 20-μm-wide Nb strip lying across of a 100-μm-wide QAHI Hall-bar device.** The magnetic field was applied perpendicular to the film. A picture of the device is shown in the inset.

remanant and saturation magnetizations. These magnetization values are expected to only slightly increase when the temperature is further decreased from 2 K to the mK temperatures used in the transport experiments reported here, since 2 K is already significantly below the Curie temperature (Fig. S11). Hence, for all relevant conditions in the experiments presented here (temperature and applied magnetic field), the magnetic induction originating from the QAHI film is negligible compared to the applied magnetic fields as well as to the critical field of niobium.

To directly confirm that the Nb superconductivity is not affected by the magnetization of the QAHI film, we also measured the SC properties of a 20-μm-wide Nb strip lying across a 100-μm-wide QAHI Hall-bar device. The inset of Fig. S12 shows a picture of the device, and the main panel shows a typical magnetic-field dependence of the Nb-strip resistivity, which is similar to that of our Nb finger electrodes on the InP substrate. We found no evidence for weakened

superconductivity in this Nb strip. Hence, the potentially detrimental effect of the QAHI film on the Nb superconductivity can safely be neglected.

Supplementary Note 14   Magnetic-field dependence of the extrinsic contact resistance

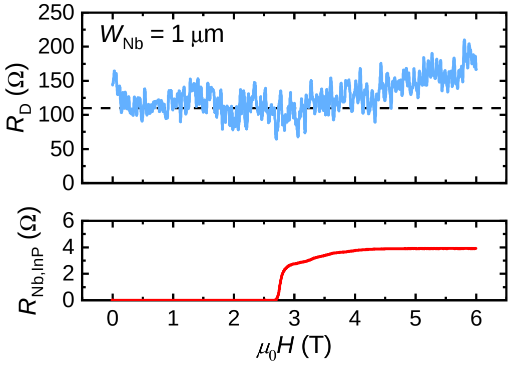

Figure S13: **Magnetic-field dependence of R_D for the 1-μm-wide Nb electrode of device G, shown together with $R_{Nb,InP}$.** The value of R_D, measured at 25 mK with $I_{RMS} = 1$ nA, remained essentially unchanged up to about 4.5 T, above which R_D starts to increase due to the breakdown of the QAH effect. The size of $R_{RNb,InP}$ is much smaller than the noise level in R_D, making its contribution above 3 T to be hardly visible.

In our analysis of ΔR_D, we assumed that the extrinsic contact resistance $R_{contact}$ due to an imperfect Nb-QAHI interface included in Eq. 1 of the main text remains unchanged across the superconducting transition of the Nb electrode. To verify this assumption, we measured R_D for a 1-μm-wide Nb electrode (device G) together with $R_{Nb,InP}$ of this finger, and the results are shown in Fig. S13. In this wide electrode, we did not observe any noticeable change in R_D across the SC transition at around ∼2.8 T within the noise level of about 50 Ω. This result justifies our calculation of ΔR_D using Eq. 1 of the main text (assuming a constant $R_{contact}$ across H_{c2}) within an uncertainty of about 50 Ω.

Supplementary Note 15 Estimation of errors

The ΔR_{D} values shown in Fig. 3b of the main text contain uncertainties, which are indicated with error bars. The main source of the uncertainty comes from the assumption that R_{contact} is constant across the SC transition, which is justified within the uncertainty of 50 Ω as discussed in the previous section. The other source of errors is the uncertainties in the determination of the values of $R_{\mathrm{D}}(H < H_{\mathrm{c2}})$ and $R_{\mathrm{D}}(H > H_{\mathrm{c2}})$. Here, the measurement noise of about 3 Ω gives one contribution. An additional contribution occurs when the observed $R_{\mathrm{D}}(H > H_{\mathrm{c2}})$ is constantly increasing, as was the case for our samples C and D shown in Fig. S7; for these samples, we considered extra errors of 16 Ω and 24 Ω, respectively. These considerations lead to the estimated total errors of 56 Ω (devices A, B, E), 72 Ω (device C), and 80 Ω (device D).

Supplementary Note 16 Estimation of the induced SC gap on the QAHI surface

By identifying ξ_{CAR} as the induced SC coherence length, one can try to infer the induced SC gap Δ_{ind} in the QAHI surface. Since the surface-state mean free path ℓ_{mfp} of our QAHI fims is unknown, we take 5 nm as a typical value for the surface of a TI device [20]. Using $v_F = 4 \times 10^5$ m/s [21] with the same dirty-limit formula as for Nb, we obtain $\Delta_{\mathrm{ind}} \approx$ 0.04 meV, which is only 3% of the Nb gap. This Δ_{ind} is probably a lower bound, since other effects of proximate Nb, such as v_F renormalization and screening of charge impurities, would make ℓ_{mfp} longer. Nevertheless, considering the presence of ferromagnetism, a small Δ_{ind} is reasonable. Note that if the 2D surface is so disordered that only puddles of metallic regions are induced, such a patchy system cannot

support CAR/CT processes which require superconducting coherence.

References:

1. Kawamura, M. et al. Current-driven instability of the quantum anomalous Hall effect in ferromagnetic topological insulators. Phys. Rev. Lett. **119**, 016803 (2017).

2. Bestwick, A. J. et al. Precise quantization of the anomalous hall effect near zero magnetic field. Phys. Rev. Lett. **114**, 187201 (2015).

3. Kou, X. et al. Scale-invariant quantum anomalous hall effect in magnetic topological insulators beyond the two-dimensional limit. Phys. Rev. Lett. **113**, 137201 (2014).

4. Fox, E. J. et al. Part-per-million quantization and current-induced breakdown of the quantum anomalous Hall effect. Phys. Rev. B **98**, 075145 (2018).

5. Fijalkowski, K. M. et al. Quantum anomalous Hall edge channels survive up to the Curie temperature. Nat. Commun. **12**, 5599 (2021).

6. Lippertz, G. et al. Current-induced breakdown of the quantum anomalous Hall effect. Phys. Rev. B **106**, 045419 (2022).

7. Datta, S. Electronic Transport in Mesoscopic Systems. Cambridge Studies in Semiconductor Physics and Microelectronic Engineering (Cambridge University Press, 1995).

8. Lambert, C. J. & Raimondi, R. Phase-coherent transport in hybrid superconducting nanostructures. J. Phys.: Condens. Matter **10**, 901–941 (1998).

9. Galambos, T. H., Ronetti, F., Hetényi, B., Loss, D. & Klinovaja, J. Crossed Andreev reflection in spin-polarized chiral edge states due to the Meissner effect. Phys. Rev. B **106**, 075410 (2022).

10. Hatefipour, M. et al. Induced superconducting pairing in integer quantum Hall edge states. Nano Lett. **22**, 6173–6178 (2022).

11. Yu, R. et al. Quantized anomalous Hall effect in magnetic topological insulators. Science **329**, 61–64 (2010).

12. Zhang, R.-X., Hsu, H.-C. & Liu, C.-X. Electrically tunable spin polarization of chiral edge modes in a quantum anomalous Hall insulator. Phys. Rev. B **93**, 235315 (2016).

13. Shen, J. et al. Spectroscopic fingerprint of chiral Majorana modes at the edge of a quantum anomalous Hall insulator/superconductor heterostructure. Proc. Nat. Acad. Sci. **117**, 238–242 (2020).

14. Lee, G.-H. et al. Inducing superconducting correlation in quantum Hall edge states. Nat. Phys. **13**, 693–698 (2017).

15. Gül, O. et al. Andreev reflection in the fractional quantum Hall state. Phys. Rev. X **12**, 021057 (2022).

16. Legg, H. F., Loss, D. & Klinovaja, J. Metallization and proximity superconductivity in topological insulator nanowires. Phys. Rev. B **105**, 155413 (2022).

17. Rüßmann, P. & Blügel, S. Proximity induced superconductivity in a topological insulator. arXiv:2208.14289 (2022).

18. Wang, J., Zhou, Q., Lian, B. & Zhang, S.-C. Chiral topological superconductor and half-integer conductance plateau from quantum anomalous Hall plateau transition. Phys. Rev. B **92**, 064520 (2015).

19. Saito, K. Critical field limitation of the niobium superconducting rf cavity. In The 10th Workshop on RF Superconductivity, Tsukuba, Japan, 583–587 (2001).

20. Ghatak, S. et al. Anomalous Fraunhofer patterns in gated Josephson junctions based on the bulk-insulating topological insulator $BiSbTeSe_2$. Nano Lett. **18**, 5124–5131 (2018).

21. Yoshimi, R. et al. Dirac electron states formed at the heterointerface between a topological insulator and a conventional semiconductor. Nat. Mater. **13**, 253–257 (2014).
